# Novel Siloxane Derivatives as Membrane Precursors for Lactate Oxidase Immobilization

**DOI:** 10.3390/s23084014

**Published:** 2023-04-15

**Authors:** Darya V. Vokhmyanina, Olesya E. Sharapova, Ksenia E. Buryanovataya, Arkady A. Karyakin

**Affiliations:** Chemistry Faculty of M.V. Lomonosov, Moscow State University, 119991 Moscow, Russia

**Keywords:** lactate biosensor, Prussian blue, siloxane, immobilization from water–organic mixtures

## Abstract

We report new enzyme-containing siloxane membranes for biosensor elaboration. Lactate oxidase immobilization from water–organic mixtures with a high concentration of organic solvent (90%) leads to advanced lactate biosensors. The use of the new alkoxysilane monomers—(3-aminopropyl)trimethoxysilane (APTMS) and trimethoxy[3-(methylamino)propyl]silane (MAPS)—as the base for enzyme-containing membrane construction resulted in a biosensor with up to a two times higher sensitivity (0.5 A·M^−1^·cm^−2^) compared to the biosensor based on (3-aminopropyl)triethoxysilane (APTES) we reported previously. The validity of the elaborated lactate biosensor for blood serum analysis was shown using standard human serum samples. The developed lactate biosensors were validated through analysis of human blood serum.

## 1. Introduction

Lactate is considered as a marker for glycolysis, the anaerobic glucose metabolism, which makes it a useful metabolite for both clinical diagnostics and sports medicine. The lactate dynamics in blood was shown to be a predictor of death from shock in 1964 [1] and can be used with this aim for various hypoxia-caused diseases [2,3], including COVID-19 [4]. Sports medicine requires monitoring of blood lactate for both training and evaluating the so-called “lactate threshold” indicating the sportsperson’s physical training level [5]. Lactate is also a fermentation byproduct and can be used as a marker for food naturalness [6].

All these applications assume the analysis of complex objects of a biological nature such as blood or food samples. Thus, lactate represents a relevant target for biosensorics implying usage of highly selective biomolecules for analyte recognition. Starting from the early 1980s, lactate oxidase (LOx) became the terminal enzyme for lactate biosensors’ elaboration [7,8]. The first lactate biosensors utilized oxygen sensing by the Clark electrode [7]. However, this approach suffered from the influence of the oxygen concentration in the sample on the result. Later research concentrated on using hydrogen peroxide (the byproduct of the enzyme-catalyzed reaction) detection [6,9]. For blood analysis, the most advanced approach seems to be the use of the hydrogen-peroxide low-potential reduction reaction provided by the advantageous catalyst—Prussian blue [10,11,12].

Lactate oxidase (LOx) is one of the less stable oxidases; so, the immobilization protocol is of great importance. For optimum stability and bioreaction efficiencies, the preferred host matrix must be one that isolates the biomolecule, protecting it from self-aggregation, while providing essentially the same local aqueous microenvironment as in the biological media [13]. The use of one of the most known matrixes for enzyme immobilization—negatively charged Nafion—obviously should dramatically reduce the dynamic range of the resulting biosensor because the analyte (lactate) is also negatively charged. Sol–gel membranes offer a better way to immobilize LOx within their porous matrix due to the simple sol–gel processing conditions and the possibility of tailoring [14,15]. This approach is unique because immobilization is based on the siloxane polymer growing around the biomolecule. The entrapped enzyme remains accessible for analytes because of the porous nature of the sol–gel network [16]. ORMOSILS (organically modified silane precursors) showed promising results in preserving the native activity of biomolecules compared to inorganic sol–gel glasses. The introduction of various functional groups such as amino, glycidoxy, vinyl, etc., into alkoxide monomers leads to organically modified sol–gel membranes. ORMOSILS provide a versatile way to prepare modified sol–gel materials. The intrinsic properties of sol–gel matrixes (e.g., porosity, surface area, polarity, and rigidity) are highly dependent on the progress of hydrolysis and condensation reactions as well as the choice of monomers, water to monomer molar ratios, solvents, etc. [14,17].

Uniform gel membranes should be deposited from diluted alkoxysilane solutions (<3–5%). Since the optimal amount of water is the one required for hydrolysis of alkoxysilane, the H_2_O content in the membrane-casting solution of trialkoxysilanes should be less than 9–15%. Thus, water–organic mixtures with a high content of organic solvent must be used for the enzyme immobilization in a siloxane gel membrane. Using a previously reported immobilization protocol from water–organic mixtures with a high content of organic solvents [10] made it possible to obtain a reusable biosensor on the base of lactate oxidase [11].

Thus, with the appropriate use of ORMOSILS together with the advanced protocol of enzyme immobilization from water–organic mixtures, one can alter the ultimate physicochemical properties of the sensing material produced and may elaborate new advantageous biosensors with improved analytical figures of merit for clinical applications. In this regard, the search for new derivatives of alkoxysilanes as membrane-forming components for the immobilization of lactate oxidase seems necessary to elaborate advanced biosensors. We have investigated siloxane monomers with various substituents in order to obtain the advantageous analytical performance of lactate biosensors. From the seven examined siloxanes, MAPS, MTES, and ETES were never reported as membrane-forming agents for enzyme immobilization, and VTMS was used for glucose oxidase immobilization [18], Supporting Information], VTES was reported as a membrane-forming agent for LOx immobilization [10], but no analytical characteristics were published. APTMS was used to develop the lactate biosensor with LOx immobilized on a layer of siloxane gel [19]. The analytical performances for this biosensor were a linear range of 5 × 10^−5^–5 × 10^−3^ M and a detection limit of 1 × 10^−5^ M. APTES is the most widely used from the examined precursors, and the biosensor based on APTES was prepared as described previously [11]. The use of a membrane based on a new siloxane monomer (MAPS) for the immobilization of lactate oxidase made it possible to obtain a biosensor with twice the sensitivity compared to a biosensor based on the most widely used siloxane in biosensors, APTES.

## 2. Materials and Methods

### 2.1. Reagents and Objects of Analysis

Experiments were carried out with Milli-Q water (18.2 MΩ·cm). Inorganic salts, hydrogen peroxide (30% solution), potassium lactate (60% solution), (3-aminopropyl)triethoxysilane (APTES) (99%), (3-aminopropyl)trimethoxysilane (APTMS) (97%), trimethoxy[3-(methylamino)propyl]silane (MAPS) (95%), vinyltrimethoxysilane (VTMS) (97%), triethoxyvinylsilane (VTES) (97%), triethoxymethylsilane (MTES) (99%), triethoxy(ethyl)silane (ETES) (96%), and organic solvents were obtained from Sigma-Aldrich (Burlington, MA, USA) or Reachim (Moscow, Russia) at the highest purity and used as received.

Lactate oxidase (LOx, EC1.1.3.2) from Pediococcus species (Sorachim, Lausanne, Switzerland) was used in the form of a lyophilized protein with a declared activity of 32.8 U/mg. Standardized human serum samples were obtained from Spinreact (Girona, Spain).

Planar three-electrode hydrogen peroxide sensors (i.e., a Prussian-blue-modified carbon working electrode, a carbon counter electrode, and a Ag/AgCl reference electrode) were purchased from Rusens LTD (Moscow, Russia). Sensor performance characteristics in batch-regime mode showed a sensitivity of 0.7 ± 0.1 A·M^−1^·cm^−2^ and a lower detection limit of 5 × 10^−7^ M.

### 2.2. Biosensor Preparation

Lactate-oxidase-containing membrane-casting mixtures were made by suspending an aqueous enzyme solution (10 mg/mL) in isopropanol containing siloxane (APTES, APTMS, MAPS, VTMS, VTES, MTES, or ETES). Siloxane solutions in isopropyl alcohol were prepared from commercial stock solution immediately before use and were used for no more than 6 h. The final concentrations in the water–isopropanol mixture were lactate oxidase 1 mg/mL, siloxane 0.1–3 _vol_%, and water 10 _vol_%. The mixture (2 µL) was drop cast onto a rough screen-printed Prussian-blue-modified carbon working electrode (Rusens LTD, Moscow, Russia) straightaway after preparation and dried in a refrigerator (4 °C) for 12 h. The enzyme-containing membrane was formed on the electrode surface after solvent evaporation and the polycondensation process. The resulted biosensors were stored in a dry state in a sealed envelope at 4 °C between the measurements.

### 2.3. Electrochemical Measurements

Electrochemical investigations were carried out using a PalmSens 4 potentiostat (PalmSens BV, Houten, The Netherlands). All the applied potentials mentioned in the paper refer to the internal Ag pseudo-reference electrode (potential of 0.25 V versus an SHE). The response of the biosensors towards the lactate was evaluated by chronoamperometry in batch mode. All the measurements were performed in 0.05 M phosphate buffer solution with 0.1 M KCl at pH 6.0 and at an applied potential of 0.0 mV. The calibration curves of lactate were separately obtained with three different biosensors (using each biosensor for all the concentration values tested). The operational stability of the elaborated lactate biosensor was investigated in 0.25 mM of lactate in batch mode upon stirring. The time of 50% loss of the initial signal was used to characterize the operational stability. The residual sensitivity was determined by comparing the analytical characteristics obtained immediately after the biosensor construction and during 12 months of storage in a dry state in a sealed envelope at 4 °C.

### 2.4. Control Serum Analysis

Standardized human serum samples with normal and pathologic analyte concentrations were prepared as described in the product instructions by reconstituting lyophilized human serum with 5 mL of distilled water. The prepared human serum samples were diluted 50 times by phosphate buffer solution prior to analysis. The lactate amperometric detection was carried out in the flow injection mode using the calibration curve obtained with standard solutions in a range of 0.01–0.1 mM.

## 3. Results and Discussion

A lactate biosensor was elaborated by lactate oxidase immobilization in a siloxane-based membrane on the top of the Prussian-blue-modified working electrode surface of the screen-printed three-electrode structures. To obtain uniform and stable membranes, the sol–gel procedure had a low water content of 10% in the water–isopropanol mixture. The latter was chosen in accordance with the optimum of lactate oxidase surviving in the water–organic mixtures known from previous works [10].

Siloxane-based membranes are promising materials for lactate oxidase immobilization due to the absence of a negative charge, which leads to the absence of electrostatic barriers for substrate diffusion to the immobilized enzyme [20]. As the structure of the monomer affects the polycondensation process rate and the enzyme environment in the resulting membrane, various siloxanes were investigated as matrices for lactate oxidase immobilization (Table 1). All the data in Table 1 and below are our original data, unless labeled otherwise with the corresponding reference.

The content of the siloxanes in the water–organic mixture used for enzyme immobilization was optimized in the range of 0.1–3 _vol_% to achieve the highest sensitivity of the resulting lactate-sensitive electrode; the optimal amounts are presented in Table 1. The triethoxysiloxane and trimethoxysiloxane containing the same substituent, vinyl, which is not involved in hydrolysis, were used for enzyme immobilization (VTES and MTES in Table 1). Even if the monomers were distinguished only by the ester groups (methoxy- or ethoxy-), the resulting biosensors had different analytical performances. The optimal monomer concentration in the membrane-casting mixture also varied depending on the ester group. This may be due to the different hydrolysis reaction rates when using ethoxy- or methoxy- derivatives [21] leading to different membrane density and enzyme microenvironments. The structure of non-hydrolyzed group also had an impact on the biosensor characteristics. Biosensors based on derivatives with short alkyl groups (methyl- or ethyl-) were characterized by relatively low sensitivity (Figure S1). The most sensitive biosensors were obtained using 3-aminopropyl-siloxanes: APTES, APTMS, and MAPS. The biosensors based on VTES also showed good sensitivity (Figure S2).

The level of L-lactate in the blood normally ranges from 0.5 to 2.2 mmol/L [22]. During intense physical activity, this index can reach 12–25 mmol/L [23]. The standard protocol for clinical lactate analyzers demands a fiftyfold sample dilution. Thus, clinical diagnostics require a lactate biosensor with a linear range from 10 to 500 µM. Using MAPS, VTMS, ETES, and MTES provided biosensors with the relevant characteristics (see Table 1). Cyclic voltammograms of the lactate biosensors demonstrated a catalytic shape in the lactate solutions with concentrations up to 1 mM (Figure S3).

**Figure 1:**
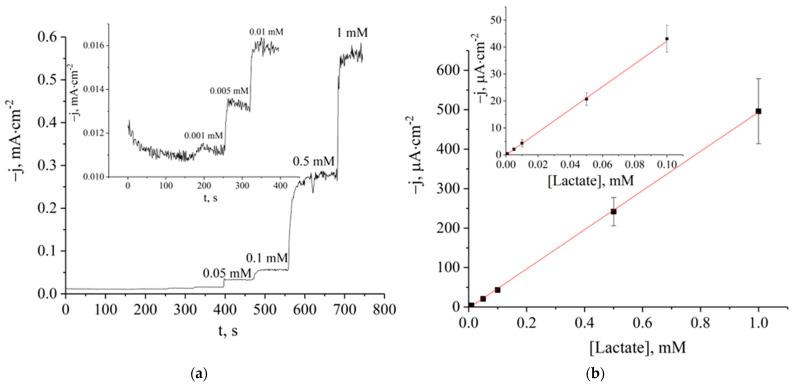
(**a**) The calibration curve for the lactate biosensor with a 1 _vol_% of MAPS in an enzyme-containing membrane solution; (**b**) calibration graph for the lactate biosensor in the batch mode (E = 0.00 V, phosphate buffer, pH 6.0). The zoomed initial parts of the correspondent dependencies are shown in the insets.

Figure 1a displays an example of the amperometric responses of the elaborated biosensor to various lactate injections in batch mode. The biosensor was made by lactate oxidase immobilization in the sol of MAPS (1% content in the mixture) over the Prussian-blue-modified electrode. There was no signal decrease during the measurement even at high lactate levels. The calibration curve for the membrane composition based on data for five different biosensors is shown in Figure 1b. A linear response was observed in a wide lactate concentration range of 1–1000 µM. The sensitivity evaluated as the slope of the calibration graph was of 0.5 A·M^−1^·cm^−2^, which is almost two times higher than for the biosensors based on APTES made by the technique elaborated previously [11].

**Figure 2:**
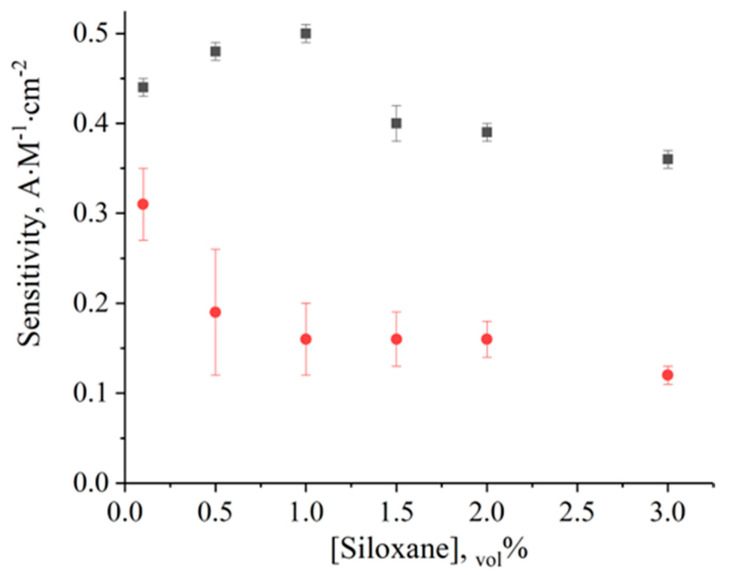
Dependence of the lactate biosensor sensitivity on the siloxane concentration in the membrane solution. Siloxanes: MAPS (■) and APTMS (●).

The analytical performances of the siloxane-based biosensors depended on the siloxane content in the casting mixture as shown in Figure 2. An increase in the siloxane concentration should lead to an increase in the membrane density and hinder the substrate diffusion to the enzyme, as seen for (3-aminopropyl)trimethoxysilane-based membranes [11]. At the same time, biosensors based on MAPS showed optimum sensitivity with siloxane concentrations in the range of 1–1.5% possibly due to the optimal enzyme environment in such membranes.

The relative selectivity of the developed biosensor to various interferences is of great interest. In general, the selectivity of the Prussian-blue-based biosensors, which operated due to hydrogen peroxide reduction, to the so called reductants (ascorbate, urate, paracetamol), was provided by the low operation potential [10]. The selectivity of the biosensors based on lactate oxidase to saccharides and hydroxy acids was due to the high selectivity of the enzyme [24].

**Figure 3:**
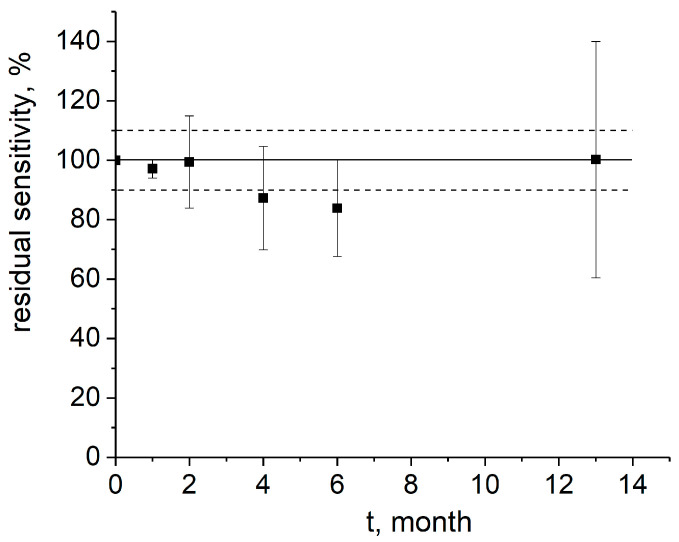
Residual sensitivity of the lactate biosensor after storage in the refrigerator (4 °C).

The storage stability is also of great importance. Figure 3 shows that no significant sensitivity loss was observed during one year of storage in a refrigerator.

A potential limitation of the developed biosensor is the half-inactivation time (operational stability), the same as for the APTES-based biosensor: 4 h in 0.25 mM lactate upon stirring (Figure S4). It can be improved by using stabilized layers of Prussian blue as suggested in [25] or by using Nafion together with siloxane as in [26] during subsequent studies.

The biosensor was validated in the course of analysis of standardized human serum with normal and pathologic lactate concentrations. The data obtained using the FIA system equipped with the elaborated lactate biosensor were in good agreement with the levels of lactate shown in the sample passport data (Table 2). Hence, the elaborated biosensor is valid for lactate detection in blood serum.

Thus, a new membrane-forming compound was found, allowing elaboration of advantageous biosensors for lactate detection, which can be used for laboratory serum analysis for both clinical and sports medicine.

## 4. Conclusions

The use of water–organic mixtures with a high concentration of organic compounds makes it possible to use a wide range of ORMASILS as a membrane-forming component. Both hydrolyzable (methoxy- or ethoxy-) and non-hydrolyzable substituents affect the analytical performance of the obtained biosensors. The choice of the monomer used and its concentration in the membrane-casting solution can lead to biosensors with attractive characteristics. Thus, it was shown that a lactate biosensor based on 1% MAPS had a sensitivity of 0.5 A·M^−1^·cm^−2^ with a detection limit of 0.5 μM. The biosensor exhibited an appropriate stability and an excellent selectivity and may find an application in clinical analysis and food quality control. The use of the proposed approach with different siloxane monomers may lead to further improvement of biosensor characteristics. Moreover, interesting results can be obtained using polysiloxanes, as well as siloxane copolymers with different structures. Using molecularly imprinted polymers on the basis of different polysiloxanes in addition to the enzyme immobilization technique might provide a more appropriate enzyme microenvironment. Moreover, the proposed approach may be suitable for the immobilization of other enzymes, as was shown for GOx and the APTES-based membranes [18], especially if the enzyme substrate is negatively charged.

## Figures and Tables

**Table 1:** The analytical performance of lactate biosensors based on different siloxanes.

Membrane Forming Agent	Sensitivity, A·M^−1^·cm^−2^	LOD, M	Linear Range, µM
APTES, 1.5 _vol_%	0.28 ± 0.03	9 × 10^−7^	1–100
APTMS, 0.1 _vol_%	0.31 ± 0.04	5 × 10^−5^	50–500
MAPS, 1.0 _vol_%	0.5 ± 0.02	5 × 10^−7^	1–1000
VTMS, 1.0 _vol_%	0.26 ± 0.05	1 × 10^−6^	5–500
VTES, 0.5 _vol_%	0.44 ± 0.05	9 × 10^−7^	5–100
MTES, 2.0 _vol_%	0.13 ± 0.08	1 × 10^−6^	1–1000
ETES, 1.5 _vol_%	0.092 ± 0.008	1 × 10^−6^	5–1000

**Table 2:** Standardized human serum analysis.

Sample	Measured Data, mM	Passport Data, mM
Normal human serum	2.1 ± 0.2	1.6 ± 0.3
Pathological human serum	3.20 ± 0.03	3.2 ± 0.6

## Data Availability

Not applicable.

## Supplementary Materials

The following supporting information can be downloaded at: https://www.mdpi.com/article/10.3390/s23084014/s1, Figure S1: Dependence of the lactate biosensor sensitivity on the siloxane concentration in the membrane solution. Siloxanes: ETES (■) and MTES (●); Figure S2: Dependence of the lactate biosensor sensitivity on the siloxane concentration in the membrane solution. Siloxanes: VTMS (■) and VTES (●); Figure S3: Cyclic voltammogram for the lactate biosensor based on 1% MAPS in phosphate buffer solution with different lactate concentrations (2 mV/s); Figure S4: The operational stability of the lactate biosensors based on 1% MAPS; (E = 0.00 V, 0.25 mM lactate, phosphate buffer, pH 6.0, upon stirring).
