# Supplementary material for: Novel Siloxane Derivatives as Membrane Precursors for Lactate Oxidase Immobilization
Source: Sensors (Basel). 2023 Apr 15;23(8):4014. doi: 10.3390/s23084014 (PMC10145638; doi:10.3390/s23084014)
Supplement: Supplementary file 1 [file sensors-23-04014-s001.zip › sensors-2318067-supplementary.pdf]

Supplementary Materials

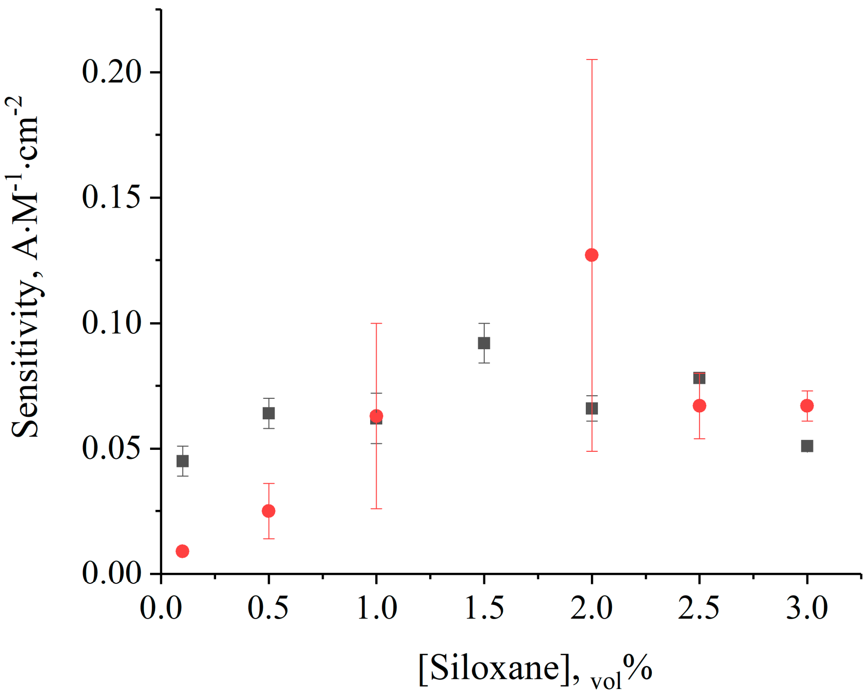

Figure S1. Dependence of the lactate biosensor sensitivity on the siloxane concentration in the membrane solution. Siloxanes: ETES (■) and MTES (●)

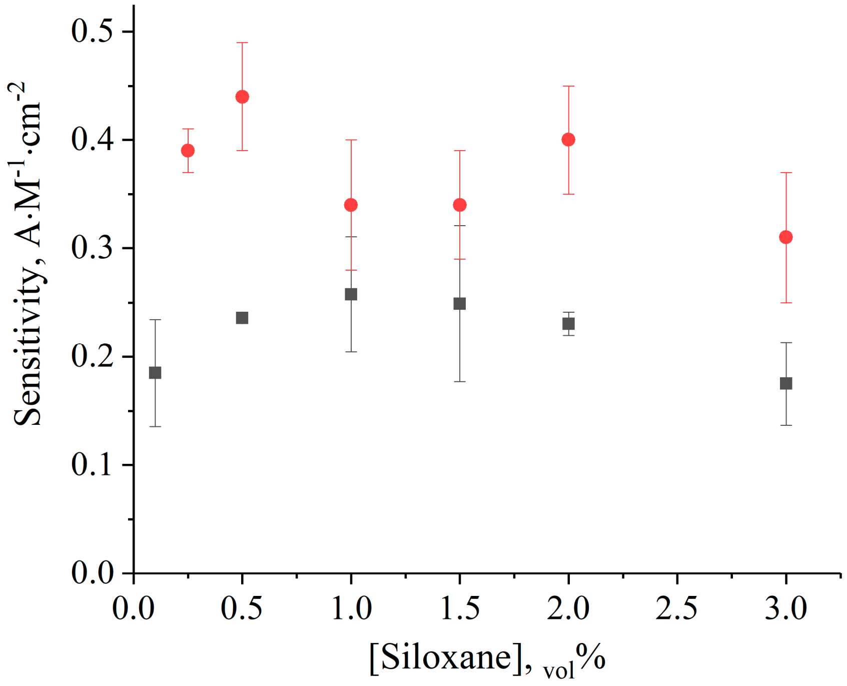

Figure S2: Dependence of the lactate biosensor sensitivity on the siloxane concentration in the membrane solution. Siloxanes: VTMS (■) and VTES (●).

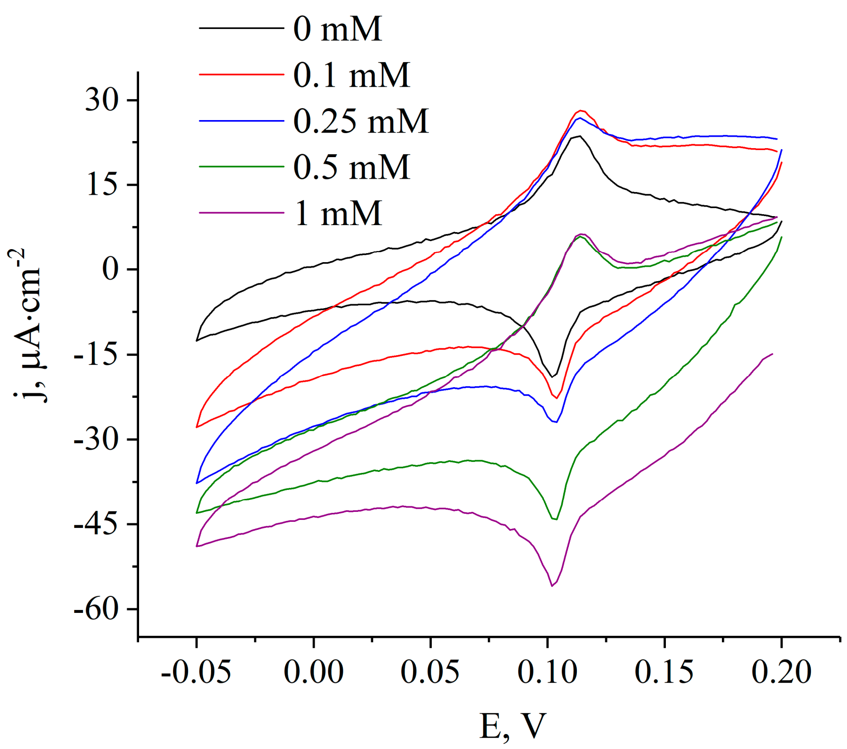

Figure S3: Cyclic voltammogram for the lactate biosensor based on 1% MAPS in phosphate buffer solution with different lactate concentrations (2 mV/s).

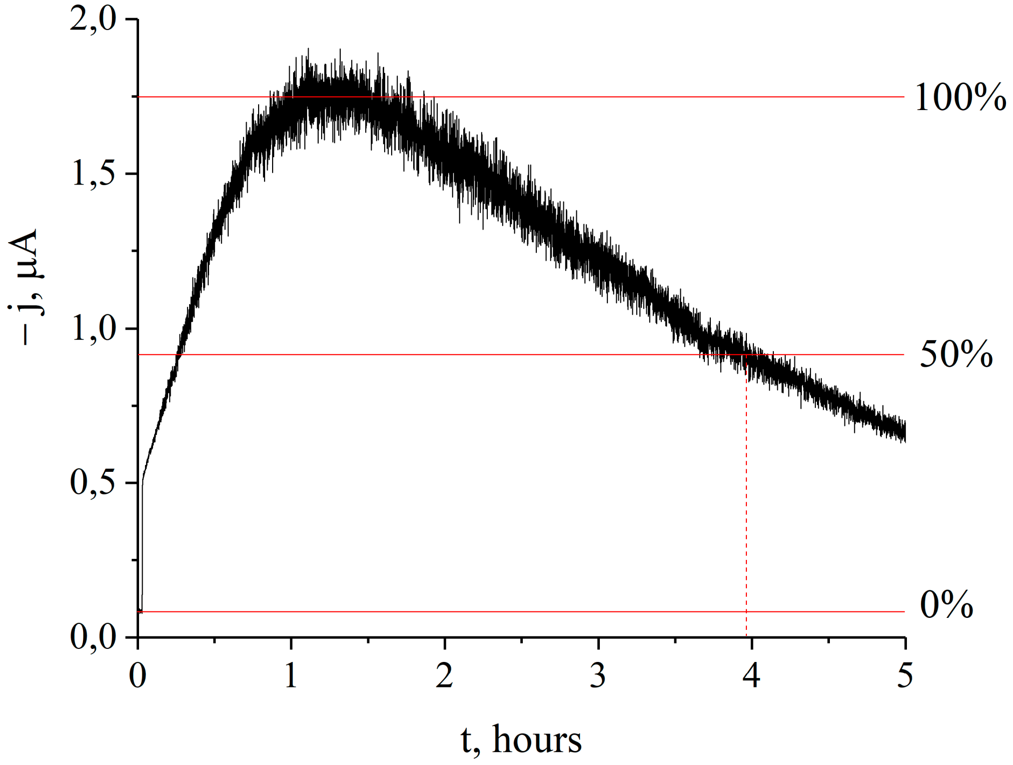

Figure S4: The operational stability of the lactate biosensors based on 1% MAPS; (E = 0.00 V, 0.25 mM lactate, phosphate buffer, pH 6.0, upon stirring).
